# Effect of long-term treatment with antioxidants (vitamin C, vitamin E, coenzyme Q10 and selenium) on arterial compliance, humoral factors and inflammatory markers in patients with multiple cardiovascular risk factors

**DOI:** 10.1186/1743-7075-7-55

**Published:** 2010-07-06

**Authors:** Marina Shargorodsky, Ortal Debby, Zipora Matas, Reuven Zimlichman

**Affiliations:** 1 Department of Endocrinology, Wolfson Medical Center, Holon, 58100, Israel; 2 Brunner Institute for Cardiovascular Research, Wolfson Medical Center, Holon, 58100, Israel; 3 Sackler School of Medicine, Tel Aviv University, Tel Aviv, Israel; 4 Department of Biochemistry, Wolfson Medical Center, Holon, 58100, Israel; 5 Department of Medicine, Wolfson Medical Center, Holon, 58100, Israel

## Abstract

**Background:**

Antioxidant supplementations have the potential to alleviate the atherosclerotic damage caused by excessive production of reactive oxygen species (ROS). The present study evaluated the effects of prolonged antioxidant treatment on arterial elasticity, inflammatory and metabolic measures in patients with multiple cardiovascular risk factors.

**Methods:**

Study participants were randomly assigned to two groups. Group 1 received oral supplementation with 2 capsules per day of Mid Life Guard, SupHerb, Israel. In each capsule vitamin C (500 mg) vitamin E (200 iu), co-enzyme Q10 (60 mg) and selenium (100 mcg), Group 2 received matching placebo(SupHerb) for 6 months.   Patients were evaluated for lipid profile, HbA1C, insulin, C-peptide, hs-CRP, endothelin, aldosterone, plasma renin activity and Homeostasis model assessment-insulin resistance (HOMA-IR). Arterial elasticity was evaluated using pulse wave contour analysis (HDI CR 2000, Eagan, Minnesota).

**Results:**

Antioxidant-treated patients exhibited significant increases in large arterial elasticity index (LAEI) as well as small arterial elasticity index (SAEI). A significant decline HbA1C and a significant increase in HDL-cholesterol were also observed. In the placebo group, significant changes in LAEI, SAEI or metabolic measures were not observed.

**Conclusions:**

Antioxidant supplementation significantly increased large and small artery elasticity in patients with multiple cardiovascular risk factors. This beneficial vascular effect was associated with an improvement in glucose and lipid metabolism as well as decrease in blood pressure.

## Background

Oxidative stress has been considered as a potential pathogenic mechanism for initiation and progression of atherosclerotic disease [1]. Excessive production of reactive oxygen species, a mediator of the oxidative stress cascade, leads to release of inflammatory cytokines, oxidation of LDL and prothrombotic state, and finally results in endothelial dysfunction and atherosclerotic vascular lesions [2-4]. Adequate dietary, enzymatic and nonenzymatic antioxidant supplementation may be effective in lowering oxidative stress. Although most trials with antioxidants in experimental models of atherosclerosis have demonstrated that this treatment is able to retard the progression of atherosclerosis, the results of clinical trials are equivocal [5]. Differences in the definition criteria of patients who are potential candidates for antioxidant treatment, type and dosage of antioxidant supplementation, as well as outcome measures may explain this variability. Since oxidative stress is activated by many cardiovascular risks factors such as hypertension, hyperglycemia, dyslipidemia and smoking, patients with multiple cardiovascular risk factors could obtain beneficial effect from antioxidant treatment [6-9]. Additionally, combinations of dietary antioxidants (vitamin C and vitamin E) with carrier in mitochondrial oxidative phosphorylation (coenzyme Q_10_) and trace elements essential for adequate function of many antioxidant enzymes (selenium) may underlie the synergism between them and amplify the positive antioxidant effect. The present study was designed to determine the effect of antioxidant supplementation with vitamin C, vitamin E, coenzyme Q_10 _and selenium on arterial compliance, inflammatory and metabolic parameters in patients with multiple cardiovascular risk factors.

## Methods

In a randomized, placebo-controlled study 70 patients with at least two cardiovascular risk factors were recruited from the hypertension outpatient clinic at E.Wolfson Medical Center for study participation. Screening procedures included physical examination, complete blood chemistry; complete blood count, urinalysis and electrocardiogram.

Cardiovascular risk factors were defined using the National Cholesterol Education Program risk factors categories: hypertension (systolic blood pressure > = 140 mm Hg and/or diastolic BP > = 90 mm Hg and/or taking antihypertensive medication); diabetes (fasting plasma glucose level > = 126 mg/dl on at least two blood samples and/or taking glucose lowering agents, hypertriglyceridemia (> = 1.7 mmol/l); low HDL cholesterol level (< 1.03 mmol/l in men and < 1.3 mmol/l in women); or current cigarette smoking.

Patients with a history of unstable angina, MI, CVA or major surgery within the six months preceding entrance to the study were excluded. Patients with unbalanced endocrine disease or any disease that might affect absorption of medications were excluded, as were patients with plasma creatinine > 2 mg/dl, elevation of liver enzymes to more that twice the upper normal limit, and electrolyte abnormalities. Patients included in the study were stabilized on their previous medical treatment in the outpatient clinic for up to three months, and an effort was made not to change treatment during the study. All concomitant medications were kept stable to prevent possible effects on the study parameters.

The study was approved by the Institutional Review Board and the patients signed a full informed consent.

Study participants were randomly assigned to two groups. Group 1 received oral supplementation with 2 capsules per day of Mid Life Guard, SupHerb, Israel. In each capsule vitamin C (500 mg) vitamin E (200 iu), co-enzyme Q10 (60 mg) and selenium (100 mcg), Group 2 received matching placebo(SupHerb) for 6 months.

### Biochemical parameters

Blood sampling for full chemistry and metabolic parameters, including fasting glucose, lipid profile, HbA1C, hs-CRP, homocysteine, endothelin, aldosterone, plasma renin activity was performed at baseline and at the end of the study.

### Arterial Elasticity Measurements

Arterial compliance measures were performed after an overnight fast and before blood sampling. Measures were performed between 8 and 10 AM, in a quiet, temperature -controlled laboratory. With the subject in a supine position, radial arterial waveforms were recorded for 30 sec. The pressure transducer amplifier system was connected to a specially designed device (Model CR-2000, Hypertension Diagnostics Inc. Eagan, MN). The passive transient response of the arterial vasculature to the initial loading conditions was determined by analyzing the diastolic portion of the pressure pulse-wave form. This technique, which has been validated for its reproducibility and used extensively [10-12], was performed with a simple noninvasive radial pulse wave recording and computer analysis of the diastolic decay. This provides separate assessment of the large artery or capacitive compliance (C1) and small artery reflective or oscillatory compliance (C2). Cardiac output and stroke volume were computed from the average waveforms. Systemic vascular resistance (SVR) is calculated as mean arterial pressure (MAP) divided by cardiac output (CO). Arterial elasticity was determined at the baseline visit and at the 3- and 6 -month on-treatment visits.

### Sample size

The primary endpoint in the present study was the paired difference in small artery elasticity (SAEI) within each group. With a sample size of n = 33 in each group, the present study was designed to have not less than 80% power to detect a true, within group difference of at least 1.2 ± 2 mL/mm Hg × 100 in SAEI in each of the two treatment groups assuming a two-tailed alpha of 0.025 (preserving to overall study alpha at 0.05).

### Statistical analysis

Analysis of data was carried out using SPSS 10.0 statistical analysis software (SPSS Inc., Chicago, IL, USA, 1999). For continuous variables, such arterial compliance parameters and biochemistry data, descriptive statistics were calculated and reported as mean ± standard deviation. Normalcy of distribution of continuous variables was assessed using the Kolmogorov-Smirnov test (cut off at p = 0.01). Categorical variables such as sex and concomitant illnesses were described using frequency distributions and are presented as frequency (%). The t-test for independent samples was used to compare continuous variables by treatment group. Categorical variables were compared by treatment group using the chi square test (exact as needed). General linear modeling was used to compare post-treatment continuous variables that differed by treatment group at baseline, including treatment group as the fixed factor and the baseline value of the modeled variable as a covariate. All tests are two-sided and considered significant at p < 0.05.

## Results

Demographic and clinical characteristics of the 70 patients with multiple cardiovascular risk factors are presented in Table 1. Group 1 included 36 patients who received oral daily supplementation with vitamin C, vitamin E, coenzyme Q_10 _and selenium. Group 2 included 34 patients who received placebo. As can be seen, both groups were similar with respect to age, sex, BMI, presence of cardiovascular risk factors, baseline blood pressure level and arterial elasticity parameters. Concomitant medications were similarly distributed in both groups at the start and end of the study.

**Table 1:** Baseline demographic and clinical characteristics of study patient

Variables	Patients received antioxidants	Patients received placebo	p value
	n = 36	**n = 34**	
Age (y)	62.17 ± 6.21	62.97 ± 5.09	0.559

Male/Female	21/13	15/21	0.678

BMI	30.10 ± 4.04	29.83 ± 5.06	0.968

Current smokers, n (%)	8 (23.53%)	10 (27.8%)	0.684

Hypertension, n (%)	25 (75.53%)	26 (72.2%)	0.902

Diabetes mellitus, n (%)	13 (38.23%)	9 (25%)	0.233

Dyslipidemia, n (%)	26 (76.47%)	28 (77.8%)	0.896

Obesity, n (%)	17 (50%)	14 (38.9%)	0.349

Family history of IHD, n (%)	26 (76.5%)	27 (75%)	0.886

Concomitant medication:			

Statins (%)	25 (73.53%)	27 (75%)	0.888

ACEIs/ARBs (%)	19(55.88%)	12 (33.33%)	0.150

Diuretics (%)	6 (17.64%)	2 (5.56%)	0.112

B-blockers (%)	19 (55.89%)	16 (44.4%)	0.338

CCB-blockers (%)	10 (29.41%)	11 (30.56%)	0.917

Aspirin (%)	13 (38.23%)	16 (44.45%)	0.598

Baseline systolic BP (mm/Hg)	125.2 ± 25.4	138.0 ± 20.8	0.203

Baseline diastolic BP (mm/Hg)	78.4 ± 11.7	75.9 ± 7.8	0.291

Baseline fasting glucose (mg/dl)	127.1 ± 52.8	118.9 ± 42.2	0.476

Baseline HbA_1_C (%)	7.1 ± 1.7	6.8 ± 1.4	0.391

Baseline total cholesterol (mg/dl)	201.5 ± 53.1	198.8 ± 50.8	0.833

Baseline LDL Cholesterol (mg/dl)	117.5 ± 43.7	118.3 ± 47.1	0.945

Baseline HDL%	22.1 ± 4.6	26.0 ± 5.4	0.004

Baseline triglycerides (mg/dl)	189.5 ± 95.0	122.8 ± 56.5	0.001

Baseline hs-CRP (mg/dl)	1.84 ± 2.9	1.35 ± 1.6	0.616

Baseline endothelin(fmol/ml)	0.7 ± 2.6	1.4 ± 4.8	0.293

Baseline homocysteine (μmol/l)	9.2 ± 2.8	8.8 ± 3.1	0.486

Baseline renin (ng/ml/hr)	1.8 ± 3.4	1.5 ± 1.8	0.695

Baseline aldosteron (pg/ml)	7.4 ± 5.6	6.7 ± 4.5	0.590

Baseline urine cathacholamines (mg/24 h)	24.74 ± 22.4	19.5 ± 12.1	0.278

### Changes in hemodynamic and arterial elasticity parameters in patients treated with antioxidants

Table 2 shows six month follow-up of hemodynamic and arterial elasticity parameters in patients received antioxidants. Systolic blood pressure (SBP) decreased significantly from 145.2 +/- 25.4 at baseline to 136.1 +/- 22.3 mmHg after 6 months of treatment (p < 0.001). Diastolic blood pressure (DBP) decreased significantly during the treatment period from 78.4 +/- 11.7 to 75.0 +/- 12.3 mmHg (p < 0.034). Heart rate did not change during the study.

**Table 2:** Change from baseline in homodynamic, arterial stiffness and metabolic variables in each group

Variables	Patients received antioxidants			Patients received placebo		
	
	baseline	6 month	p-value	baseline	6 month	p-value
Systolic BP (mm/Hg)	145.2 ± 25.4	136.1 ± 22.3	0.001	138.0 ± 20.8	135.0 ± 21.6	0.257

Diastolic BP (mm/Hg)	78.4 ± 11.7	75.0 ± 12.3	0.034	75.9 ± 7.8	75.0 ± 10.3	0.493

LAEI(ml/mm Hg × 10)	11.0 ± 4.4	12.7 ± 4.7	0.006	12.9 ± 6.2	11.3 ± 4.3	0.151

SAEI(ml/mm Hg × 100)	3.3 ± 1.9	4.7 ± 2.7	0.0001	3.6 ± 1.6	3.7 ± 1.9	0.732

SVR(dynes•sec/cm5)	1817.0 ± 751.5	1617.3 ± 305.2	0.102	1594.9 ± 351.3	1638.7 ± 311.3	0.533

Glucose(mg/dl)	127.0 ± 52	129 ± 62	0.535	118.9 ± 42	123.2 ± 55	0.496

HbA_1_C(%)	7.08 ± 1.69	6.33 ± 2.3	0.022	6.82 ± 1.44	6.86 ± 1.66	0.767

Total Cholesterol(mg/dl)	201.5 ± 53.1	180.7 ± 58.3	0.074	197.12 ± 50	186.80 ± 37.78	0.125

Triglycerides(mg/dl)	189 ± 94	170.4 ± 72	0.058	124.5 ± 56.5	127.83 ± 46.7	0.645

HDL%	22.1 ± 4	26.2 ± 10	0.022	26 ± 5.4	27.11 ± 5.5	0.212

LDL Cholesterol(mg/dl)	116.8 ± 44.4	103.4 ± 47	0.13	117.91 ± 47.9	109.0 ± 33.1	0.135

hs-CRP(mg/dl)	1.94 ± 3	0.62 ± 0.77	0.222	1.44 ± 1.59	0.95 ± 0.90	0.271

Renin (ng/ml/hr)	1.79 ± 3.4	2.27 ± 2.6	0.494	1.36 ± 1.55	1.91 ± 1.87	0.149

Aldosterone(pg/ml)	7.38 ± 5.6	8.2 ± 5.06	0.354	6.70 ± 4.5	7.00 ± 3.84	0.638

Homocysteine(μmol/l)	9.24 ± 2.82	9.19 ± 2.83	0.911	8.75 ± 3.04	9.11 ± 3.3	0.139

Ur cathacholamines(mg/24 h)	24.74 ± 21	28.5 ± 19	0.118	19.54 ± 12	22 ± 20	0.283


**Figure 1:**
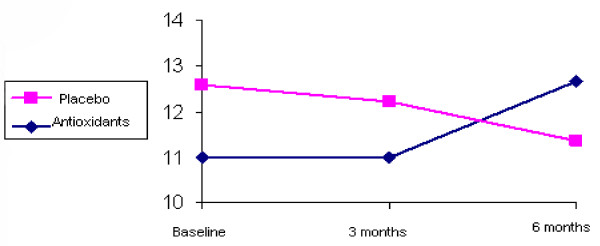
**LAEI by groups during 6-month follow-up**.

**Figure 2:**
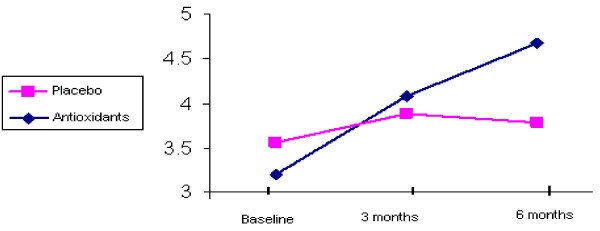
**SAEI by groups during 6-month follow-up**.

LAEI increased from 11.0 +/- 4.4 to 12.7 +/- 4.7 ml/mm Hg × 100 after 6 months of treatment (p < 0.006) (Figure 1). SAEI increased significantly during the study from 3.3 +/- 1.9 to 4.7 +/- 2.7 ml/mm Hg × 100 (p < 0.0001) (Figure 2). Although SVR decreased by 11% during the treatment period, this decrease did not reach statistical significance (p = 0.102) (Table 2).

### Changes in hemodynamic and arterial elasticity parameters in the control group

As shown in Table 2, systolic blood pressure as well as diastolic blood pressure did not change significantly during the study (p = 0.257 and p = 0.493, respectively).

Neither LAEI nor SAEI improved significantly during the treatment period. LAEI was 12.9 +/- 6.2 ml/mmHg × 100 at baseline and 11.3 +/- 4.3 ml/mmHg × 100 after 6 months of follow-up (p = 0.151) (Figure 1). SAEI was 3.6 +/- 1.5 at baseline and 3.7 +/- 1.9 ml/mmHg × 100 at the end of the study (p = 0.732) (Figure 2). SVR tended to increase during the study from 1594.7 +/- 351.3 to 1638.7 +/- 311.3 dyne × sec × cm, however this increase did not reach statistical significance (p = 0.533) (Table 2).

### Changes in metabolic and inflammatory parameters during 6-month treatment period

As shown in Table 2, levels of HbA_1_C decreased significantly from 7.08 ± 1.69% to 6.33 ± 2.3% (p = 0.022) in patients received antioxidants. Additionally, significant increase in HDL-cholesterol (p = 0.022) and marginal declines in triglycerides (p = 0.058) as well as total cholesterol levels (p = 0.074) were observed in antioxidant treated patients.

Metabolic parameters including HbA_1_C, triglycerides, total cholesterol, HDL-cholesterol and CRP did not change in placebo group during the study.

## Discussion

The present randomized, placebo controlled study demonstrates that antioxidant supplementation with vitamin C, vitamin E, coenzyme Q_10 _and selenium significantly increased large and small artery elasticity in patients with multiple cardiovascular risk factors. This beneficial vascular effect was associated with an improvement in glucose and lipid metabolism as well as significant decrease in blood pressure.

Assessment of arterial function and structure can serve as a surrogate endpoint for prediction of morbid events and for estimation of success of treatment. Pulse wave contour analysis using the modified Windkessel model is one of several noninvasive methods for estimation of arterial properties. Numerous studies performed with the HDI CR-2000 equipment have shown good correlation to age, cardiovascular risk factors and markers of disease [13]. Therapeutic interventions with medications like statins, angiotensin II receptor blocking agents as well as weight loss, have also shown improvement in LAEI and SAEI which may suggest lowering of cardiovascular risk. Nevertheless, no major prospective study associating arterial elasticity with cardiovascular events has been performed. Although PWA using the modified Windkessel model has some limitations, this method provides complementary information about vascular health.

The favorable vascular effect of antioxidants has been observed *in vitro *and in animal models of atherosclerosis [14-16]. However, data on long-term vascular impact of antioxidant supplementation in humans are limited and controversial. The findings of the present study concur with those of previous study that has shown substantial reduction in the progression of common carotid atherosclerosis during three year treatment with combined supplementation of both vitamin E and vitamin C [17]. Additionally, the beneficial effect of antioxidant supplementation on LDL oxidation and endothelial flow has been demonstrated [18,19]. Moreover several prospective randomized controlled clinical trials such as Cambridge Heart Antioxidant Study, Secondary Prevention with Antioxidants of Cardiovascular Disease in End-stage Renal Disease study and Cholesterol Lowering Atherosclerosis Study reported that the administration of antioxidants reduced the risk of cardiovascular disease [20-22]. Nevertheless, subsequent large interventional studies do not support a benefit from antioxidant supplementation [23,24]. These clinical trials have demonstrated that vitamin E alone or in combination has no effect on the risk of death or prevention of cardiovascular disease. Moreover, a dose-response meta-analysis has shown that high-dosage vitamin E supplementation was associated with a small but statistically significant increased risk for mortality [25]. The lack of benefit seen in these clinical trials does not disprove the central role of oxidative stress in atherosclerosis and justify investigating the overall clinical impact of antioxidant treatment.

Although the anti-atherogenic effect of antioxidants has been assessed in several experimental studies, the mechanisms by which these agents inhibit atherosclerosis remain to be clarified. Combined supplementation of vitamin E and C have been shown to inhibit DNA oxidation by H_2_O_2 _in human lymphocytes, to enhance endogenous plasma and tissue antioxidant defenses and restore endothelium-dependent vasoactivity [18,26,27]. Coenzyme Q_10 _which plays an essential role as an electron carrier in mitochondrial oxidative phosphorylation, improves endothelial dysfunction in diabetic patients [28]. Finally, selenium as a determinant of antioxidative glutathione peroxidase 1 expression and activity, provides significant protection of the coronary artery endothelium against damage by oxidative stress [29].

The findings of the present study concur with those of previous studies that have shown substantial reduction in blood pressure and improvement in long-term glycaemic control with oral CoQ supplementation, reduction in plasma glucose and insulin resistance with high doses of vitamin E supplementation and significant reduction in blood pressure levels with vitamin E as well as vitamin C in hypertensive patients [30-33]. Nevertheless, which particular antioxidant or combination of antioxidants is responsible for the favorable metabolic effect in the present study remains uncertain. Additionally, we cannot exclude the possibility that specific antioxidant combination which was used in the present study, has a contributory effect of on blood pressure, glucose and lipid homeostasis as well as on improvement of vascular elasticity.

In the present study, we did not observe significant changes in humoral factors such as homocystein, endothelin, aldosterone and renin in subjects received antioxidant supplementation. Levels of urine cathecholamines also did not change during the treatment period. These findings emphasize a previously published data which have shown that patophysiologic mechanism of antioxidants action is independent of the changes in plasma concentration of blood pressure modulators, such as renin, aldosterone, endothelin [34], although the precise mechanism for antioxidant action on the vasculature remains to be elucidated.

Our study has several limitations. First, the present study contains relatively small number of participants and larger studies are required to establish the beneficial vascular effect of antioxidant supplementation. Second, we did not measure plasma levels of the antioxidants which would have added the information regarding treatment compliance and would have elucidated the pathophysiology for vascular action of antioxidants. Furthermore, since the present study has focused on patients with multiple cardiovascular risk factors, the application of our findings to other patient populations remains uncertain.

## Conclusion

We have demonstrated that combined antioxidant supplementation with vitamin C, vitamin E, coenzyme Q_10 _and selenium has beneficial effect on glucose and lipid metabolism, blood pressure and arterial compliance in patients with multiple cardiovascular risk factors. The findings of the present study justify investigating the overall clinical impact of antioxidant treatment in this population.

## Abbreviations

CI: confidence interval; NSAIDs: non-steroidal anti-inflammatory drugs; SD: standard deviation.

## Competing interests

The authors declare that they have no competing interests.

## Authors' contributions

MS, OD, ZM and RZ contributed to the study conception and design. MS, OD and RZ were responsible for the data acquisition. RZ and OD performed the analysis and interpretation of data. ZM carried out the immunoassays. MS were responsible for review the existing literature and for writing the first draft of the paper. All authors performed a critical revision of the manuscript for important intellectual content. All authors read and approved the final manuscript.

## References

[B1] Stocker R Keaney JF Jr Role of oxidative modifications in atherosclerosis Physiol Rev 2004 84 1381 1478 10.1152/physrev.00047.2003 15383655

[B2] Schleicher E Friess U Oxidative stress, AGE, and atherosclerosis Kidney International 2007 72 S17 S26 10.1038/sj.ki.5002382 17653206

[B3] Griendling KK FitzGerald GA Oxidative stress and cardiovascular injury: Part I: basic mechanisms and *in vivo *monitoring of ROS Circulation 2003 108 1912 1916 10.1161/01.CIR.0000093660.86242.BB 14568884

[B4] Kunsch C Medford RM Oxidative stress as a regulator of gene expression in the vasculature Circ Res 1999 85 753 766 10521248 10.1161/01.res.85.8.753

[B5] Madamanchi NR Hakim ZS Runge MS Oxidative stress in atherogenesis and arterial thrombosis: the disconnect between cellular studies and clinical outcomes J Thromb Haemost 2005 3 254 267 10.1111/j.1538-7836.2004.01085.x 15670030

[B6] Maxwell SR Thomason H Sandler D LeGuen C Baxter MA Thorpe GH Jones AF Barnett AH Poor glycaemic control is associated with reduced serum free radical scavenging (antioxidant) activity in non-insulin-dependent diabetes mellitus Ann Clin Biochem 1997 34 638 644 9367001 10.1177/000456329703400607

[B7] Sanguigni V Pignatelli P Caccese D Pulcinelli FM Lenti L Magnaterra R Martini F Lauro R Violi F Increased superoxide anion production by platelets in hypercholesterolemic patients Thromb Haemost 2002 87 796 801 12038779

[B8] Beswick RA Dorrance AM Leite R Webb RC NADH/NADPH oxidase and enhanced superoxide production in the mineralocorticoid hypertensive rat Hypertension 2001 38 1107 11 10.1161/hy1101.093423 11711506

[B9] Morrow JD Frei B Longmire AW Gaziano JM Lynch SM Shyr Y Strauss WE Oates JA Roberts LJ Increase in circulating products of lipid peroxidation (F2-isoprostanes) in smokers. Smoking as a cause of oxidative damage N Engl J Med 1995 332 1198 203 10.1056/NEJM199505043321804 7700313

[B10] Zieman SJ Melenovsky V Kass DA Mechanisms, pathophysiology, and therapy of arterial stiffness Arterioscler Thromb Vasc Biol 2005 25 932 943 10.1161/01.ATV.0000160548.78317.29 15731494

[B11] Giannattasio C Mancia G Arterial distensibility in humans. Modulating mechanisms, alterations in diseases and effects of treatment J Hypertens 2002 20 1889 1899 10.1097/00004872-200210000-00001 12359960

[B12] Cohn JN Finkelstein S McVeigh G Morgan D LeMay I Robinson J Mock J Non- invasive pulse wave analysis for the detection of arterial vascular disease Hypertension 1995 26 503 508 7649589 10.1161/01.hyp.26.3.503

[B13] Zimlichman R Shargorodsky M Boaz M Duprez D Rahn D Rizzoni D Payeras AC Hamm C McVeigh G Determination of arterial compliance using blood pressure waveform analysis with the CR-2000 system: Reliability, repeatability, and establishment of normal values for healthy European population Am J Hypertens 2005 18 1 65 71 10.1016/j.amjhyper.2004.08.013 15691619

[B14] Hsich E Segal BH Pagano PJ Rey FE Paigen B Deleonardis J Hoyt RF Holland SM Finkel T Vascular effects following homozygous disruption of p47(phox): An essential component of NADPH oxidase Circulation 2000 101 1234 1236 10725280 10.1161/01.cir.101.11.1234

[B15] Chen X Touyz RM Bae Park J Schiffrin EL Antioxidant effects of vitamins C and E are associated with altered activation of vascular NADPH oxidase and superoxide dismutase in stroke-prone SHR Hypertension 2001 38 606 611 10.1161/hy09t1.094005 11566940

[B16] Wu BJ Kathir K Witting PK Beck K Choy K Li C Croft KD Mori TA Tanous D Adams MR Lau AK Stocker R Antioxidants protect from atherosclerosis by a heme oxygenase-1 pathway that is independent of free radical scavenging J Exp Med 2006 203 1117 1127 10.1084/jem.20052321 16606673 PMC2118288

[B17] Salonen JT Nyyssönen K Salonen R Lakka HM Kaikkonen J Porkkala-Sarataho E Voutilainen S Lakka TA Rissanen T Leskinen L Tuomainen TP Valkonen VP Ristonmaa U Poulsen HE Antioxidant Supplementation in Atherosclerosis Prevention Study (ASAP): a randomized trial of the effect of vitamins E and C on 3-year progression of carotid atherosclerosis J Intern Med 2000 248 377 386 10.1046/j.1365-2796.2000.00752.x 11123502

[B18] Plotnick GD Corretti MC Vogel RA Effect of antioxidant vitamins on the transient impairment of endothelium-dependent brachial artery vasoactivity following a single high-fat meal JAMA 1997 278 1682 1686 10.1001/jama.278.20.1682 9388088

[B19] Devaraj S Jialal I Low-density lipoprotein postsecretory modification, monocyte function, and circulating adhesion molecules in type 2 diabetic patients with and without macrovascular complications: the effect of α-tocopherol supplementation Circulation 2000 102 191 196 10889130 10.1161/01.cir.102.2.191

[B20] Stephens NG Parsons A Schofield PM Kelly F Cheeseman K Mitchinson MJ Randomised controlled trial of vitamin E in patients with coronary disease: Cambridge Heart Antioxidant Study (CHAOS) Lancet 1996 347 781 786 10.1016/S0140-6736(96)90866-1 8622332

[B21] Boaz M Smetana S Weinstein T Matas Z Gafter U Iaina A Knecht A Weissgarten Y Brunner D Fainaru M Green MS Secondary prevention with antioxidants of cardiovascular disease in endstage renal disease (SPACE): randomised placebo-controlled trial Lancet 2000 356 1213 1218 10.1016/S0140-6736(00)02783-5 11072938

[B22] Brown BG Zhao XQ Chait A Fisher LD Cheung MC Morse JS Dowdy AA Marino EK Bolson EL Alaupovic P Frohlich J Albers JJ Simvastatin and niacin, antioxidant vitamins, or the combination for the prevention of coronary disease N Engl J Med 2001 345 1583 1592 10.1056/NEJMoa011090 11757504

[B23] Yusuf S Dagenais G Pogue J Bosch J Sleight P Vitamin E supplementation and cardiovascular events in high-risk patients. Heart Outcomes Prevention Evaluation Study Investigators N Engl J Med 2000 342 154 160 10.1056/NEJM200001203420302 10639540

[B24] Collaborative Group of the Primary Prevention Project Low-dose aspirin and vitamin E in people at cardiovascular risk: a randomized trial in general practice Lancet 2001 357 89 95 10.1016/S0140-6736(00)03539-X 11197445

[B25] Miller ER Pastor-Barriuso R Dalal D Riemersma RA Appel LJ Guallar E Meta-analysis: high-dosage vitamin E supplementation may increase all-cause mortality Ann Intern Med 2005 142 37 46 15537682 10.7326/0003-4819-142-1-200501040-00110

[B26] Brennan LA Morris GM Wasson GR Hannigan BM Barnett YA The effect of vitamin C or vitamin E supplementation on basal and H2O2-induced DNA damage in human lymphocytes Br J Nutr 2000 84 195 202 10.1079/096582197388680 11029970

[B27] Dusinská M Kazimírová A Barancoková M Beno M Smolková B Horská A Raslová K Wsólová L Collins AR Nutritional supplementation with antioxidants decreases chromosomal damage in humans Mutagenesis 2003 18 371 376 10.1093/mutage/geg002 12840111

[B28] Watts GF Playford DA Croft KD Ward NC Mori TA Burke V Coenzyme Q_10 _improves endothelial dysfunction of the brachial artery in type II diabetes mellitus Diabetologia 2002 45 420 426 10.1007/s00125-001-0760-y 11914748

[B29] Miller S Walker SW Arthur JR Nicol F Pickard K Lewin MH Howie AF Beckett GJ Selenite protects human endothelial cells from oxidative damage and induces thioredoxin reductase Clin Sci (Lond) 2001 100 5 543 50 10.1042/CS20000299 11294695

[B30] Hodgson JM Watts GF Playford DA Burke V Croft KD Coenzyme Q_10 _improves blood pressure and glycaemic control: a controlled trial in subjects with type 2 diabetes European Journal of Clinical Nutrition 2002 56 1137 1142 10.1038/sj.ejcn.1601464 12428181

[B31] Manning PJ Sutherland WH Walker RJ Williams SM De Jong SA Ryalls AR Berry EA Effect of high-dose vitamin E on insulin resistance and associated parameters in overweight subjects Diabetes Care 2004 27 2166 2171 10.2337/diacare.27.9.2166 15333479

[B32] Boshtam M Rafiei M Sadeghi K Sarraf-Zadegan N Vitamin E can reduce blood pressure in mild hypertensives Int J Vitam Nutr Res 2002 72 309 314 10.1024/0300-9831.72.5.309 12463106

[B33] Duffy SJ Gokce N Holbrook M Huang A Frei B Keaney JF Jr Vita JA Treatment of hypertension with ascorbic acid Lancet 1999 354 2048 2049 10.1016/S0140-6736(99)04410-4 10636373

[B34] Rodrigo R Prat H Passalacqua W Araya J Bächler JP Decrease in oxidative stress through supplementation of vitamins C and E is associated with a reduction in blood pressure in patients with essential hypertension Clin Sci (Lond) 2008 114 10 625 34 10.1042/CS20070343 17999638

